# A multivariate prediction model for microarray cross-hybridization

**DOI:** 10.1186/1471-2105-7-101

**Published:** 2006-03-01

**Authors:** Yian A Chen, Cheng-Chung Chou, Xinghua Lu, Elizabeth H Slate, Konan Peck, Wenying Xu, Eberhard O Voit, Jonas S Almeida

**Affiliations:** 1 Department of Biostatistics, Bioinformatics, and Epidemiology, Medical University of South Carolina, Charleston, SC, USA; 2 Center for Genomic Medicine, National Taiwan University, Taipei, Taiwan; 3 Institute of Biomedical Sciences, Academia Sinica, Taipei, Taiwan; 4 Key Laboratory of Molecular and Developmental Biology, Institute of Genetics and Developmental Biology, Chinese Academy of Sciences, Beijing, P. R China; 5 Department of Biomedical Engineering, Georgia Tech, Atlanta, GA, USA; 6 Department of Biostatistics and Applied Mathematics, University of Texas MD Anderson Cancer Center, Houston, TX, USA

## Abstract

**Background:**

Expression microarray analysis is one of the most popular molecular diagnostic techniques in the post-genomic era. However, this technique faces the fundamental problem of potential cross-hybridization. This is a pervasive problem for both oligonucleotide and cDNA microarrays; it is considered particularly problematic for the latter. No comprehensive multivariate predictive modeling has been performed to understand how multiple variables contribute to (cross-) hybridization.

**Results:**

We propose a systematic search strategy using multiple multivariate models [multiple linear regressions, regression trees, and artificial neural network analyses (ANNs)] to select an effective set of predictors for hybridization. We validate this approach on a set of DNA microarrays with cytochrome p450 family genes. The performance of our multiple multivariate models is compared with that of a recently proposed third-order polynomial regression method that uses percent identity as the sole predictor. All multivariate models agree that the 'most contiguous base pairs between probe and target sequences,' rather than percent identity, is the best univariate predictor. The predictive power is improved by inclusion of additional nonlinear effects, in particular target GC content, when regression trees or ANNs are used.

**Conclusion:**

A systematic multivariate approach is provided to assess the importance of multiple sequence features for hybridization and of relationships among these features. This approach can easily be applied to larger datasets. This will allow future developments of generalized hybridization models that will be able to correct for false-positive cross-hybridization signals in expression experiments.

## Background

Expression microarrays are powerful tools for disease diagnosis, prognosis and treatment [1], offering unparalleled insight into the function of the entire genome and the dynamic interactions among genes. Two common platforms are oligonucleotide and cDNA microarrays. Oligonucleotide microarrays are generated by either robotic deposition of pre-synthesized oligos or *in situ *synthesis of ~25-mer oligo probes ontosolid slides [2,3], while cDNA microarrays are created by spotting long strands of amplified cDNA sequences, such as expressed sequence tags (ESTs) [4].

Specific hybridization is the desired type of hybridization between a probe and the target sequence that comes from the same transcript. By contrast, cross-hybridization may occur between parts of the probe and target sequences that do not come from the same transcript as the probe. Cross-hybridization can be a significant contributor to false-positive noise in array data and is known to happen in both oligo and cDNA microarray platforms. Duplex stabilities and re-association kinetics for nucleic acid hybridization is complex, and many factors are involved. Experimental conditions such as hybridization temperature, salt concentration, viscosity of the solvents, pH value are important. Concentration, complexity, lengths, and GC contents, as well as the secondary structures of nucleic acids are also critical. A comprehensive review can be found in [5].

Hybridization in solvents is different from that on solid surfaces, and different surfaces and platforms have different properties. Several studies have been conducted to model the expression intensities using binding kinetics based on physical properties or oligo composition in the popular oligonucleotide microarrays made by Affymetrix [6-8]. Cross-hybridization is an especially severe problem for cDNA microarrays because of the lengths of the probes [9]. Because predictions of binding free energy cannot yet be achieved for longer sequences, the models developed for oligo arrays cannot be generalized to cDNA microarrays. Several univariate studies have attempted to correlate the hybridization intensities and sequence characteristics between the probe-target pair for cDNA or DNA microarrays using genomic sequences [10-13]. Most of these studies [10-12] reached the same (and non-surprising) conclusion that sequences sharing a high degree of identity have a higher chance to cross-hybridize. Another approach to studying cross-hybridization is to investigate the relationships between contiguous pairing segments and hybridization intensity [13]. All these studies acknowledged some exceptions that could not be accommodated by their univariate analyses. To the authors' knowledge, no systematic multivariate predictive modeling has been attempted for cDNA microarray hybridizations.

A field relevant to the microarray cross-hybridization issue is the design of short interfering RNA (siRNA) sequences (10 ~25 nucleotides) leading to RNA interference (RNAi). In particular, the selection of effective siRNA sequences that minimize off-target silencing effects is a topic of great interest in computational and functional genomics [14-16]. As in the field of cDNA array analysis, these reports point to the fact that more studies focus on the hybridization between short sequences (such as oligo arrays or siRNA design) rather than on cross-hybridization between long sequences.

**Figure 1:**
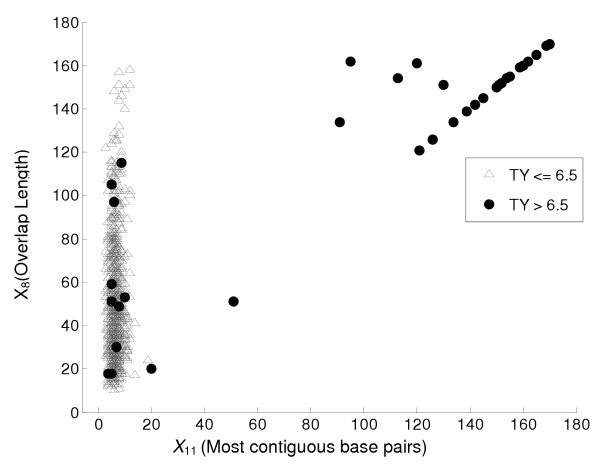
**Representation of hybridization intensities with respect to the most contiguous base pairs and overlap lengths**. Solid circles show strong hybridization intensities (*TY *> 6.5), while open triangles indicate low intensities (*TY *≤ 6.5).

Specific signal quantification is crucial for correct interpretation of microarray experiments, and probe selection has been the major task for array design in the past decade [17-29] to avoid cross-hybridization. However, the number of probes spotted on both oligo and cDNA arrays increases dramatically as the technology advances, and cross-hybridization almost becomes inevitable. A computational method validated by proper experiments to quantify platform-specific cross-hybridization is needed to derive correct quantification of sequence-specific signals. The challenge is that cross-hybridization is the result of complex interactions between multiple target and probe sequences on the arrays (see Figure 1a in Additional file 1). It seems very difficult to attack this problem in generality at this point. Therefore, as a first step toward understanding this complex phenomenon of a many-to-many relationship, we propose to investigate a simplified system with hybridization between one target and multiple probes spotted on the arrays; that is, to quantify the hybridization of one target to many probes (see Figure 1b in Additional file 1).

A dataset of CYP450 PCR products spotted on microarrays following the experimental design proposed in [30] was used for our model development. The genes in the cytochrome P450 family are known to have varying degrees of sequence similarities, thus making them good candidates for studying cross-hybridization phenomena on microarrays [11,30]. Because hybridization is influenced by sequence characteristics as well as many experimental factors, the experimental/hybridization conditions, such as target/probe concentration, salt concentration, and hybridization temperature, were kept consistent throughout this study.

The immediate goal of our current research is to identify efficacious sets of sequence features for predicting hybridization between probe-target pairs in a multivariate fashion and to determine how different factors synergistically influence hybridization. Our ultimate goal, which reaches beyond the scope of this paper, is to estimate specific hybridization features after correcting for false-positive cross-hybridization.

## Results

A dataset of CYP450 PCR products spotted on microarrays [30] was used to validate the proposed multivariate approach. Thirty-one different cDNAs from the CYP450 family (with lengths ranging from 500 to 1200 bp) were hybridized individually with each of 31 arrays. Triplicates were generated, for a total of 93 arrays. The target/probe concentrations and other experimental conditions (such as temperature and salt concentration) were constant across arrays. Details of the experiments and array manufacturing processes are described in Methods and [30].

### Preliminary analysis

**Figure 2:**
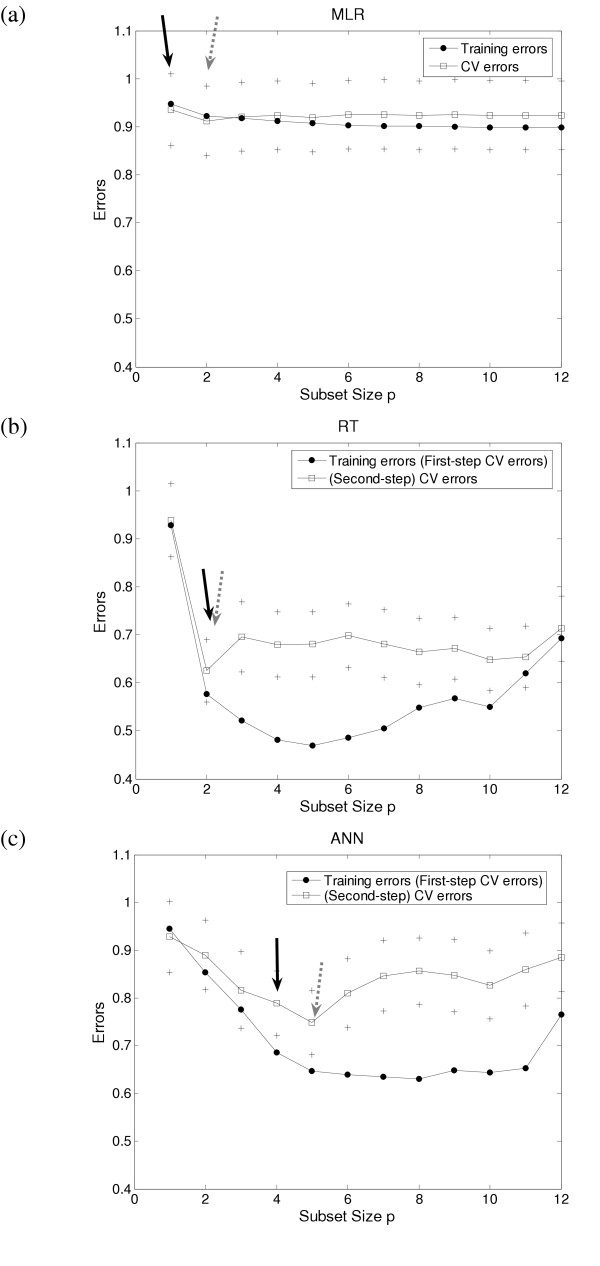
**Training and cross-validation (CV) errors of the multivariate models**. Minimum training errors (solid circles) of (a) multiple linear regressions (MLRs), (b) regression trees (RTs), and (c) artificial neural networks (ANNs) in the first CV training set decreased, while the CV errors [open squares; Equation (4)] reached the minimum (light-dotted arrows) at the subset size of 2 in (a), 2 in (b), and 5 in (c). The most parsimonious model (dark-solid arrows) within one standard error of the model with the minimum error was the model with 1 predictor for (a), 2 predictors for (b) and 4 predictors for (c). (The cross-validated variance of *TY*, for reference, is 1.43 ± 0.13).

Triplicate data were used to estimate the parameters *λ *and *α *in the generalized log transformation of the hybridization intensities [Equation (1) in Methods]. The estimated parameters were λ^
 MathType@MTEF@5@5@+=feaafiart1ev1aaatCvAUfKttLearuWrP9MDH5MBPbIqV92AaeXatLxBI9gBaebbnrfifHhDYfgasaacH8akY=wiFfYdH8Gipec8Eeeu0xXdbba9frFj0=OqFfea0dXdd9vqai=hGuQ8kuc9pgc9s8qqaq=dirpe0xb9q8qiLsFr0=vr0=vr0dc8meaabaqaciaacaGaaeqabaqabeGadaaakeaacuaH7oaBgaqcaaaa@2E70@ = 1.39*10^-20 ^and α^
 MathType@MTEF@5@5@+=feaafiart1ev1aaatCvAUfKttLearuWrP9MDH5MBPbIqV92AaeXatLxBI9gBaebbnrfifHhDYfgasaacH8akY=wiFfYdH8Gipec8Eeeu0xXdbba9frFj0=OqFfea0dXdd9vqai=hGuQ8kuc9pgc9s8qqaq=dirpe0xb9q8qiLsFr0=vr0=vr0dc8meaabaqaciaacaGaaeqabaqabeGadaaakeaacuaHXoqygaqcaaaa@2E5B@ = 1.79*10^-12^. Hybridization experiments were highly reproducible among replicates (0.94 < Spearman correlation coefficient < 0.97; see Table 1 in Additional file 1). Hybridization intensities of target 17 in all three replicates were consistently lower than others (see Figure 2a in Additional file 1). These low intensities, including specific (self-self) hybridization, indicate that systematic errors were introduced in this target sample. Therefore, the data of target 17 were excluded, and the remaining data were used to re-estimate α and λ. The re-estimated parameters were λ^
 MathType@MTEF@5@5@+=feaafiart1ev1aaatCvAUfKttLearuWrP9MDH5MBPbIqV92AaeXatLxBI9gBaebbnrfifHhDYfgasaacH8akY=wiFfYdH8Gipec8Eeeu0xXdbba9frFj0=OqFfea0dXdd9vqai=hGuQ8kuc9pgc9s8qqaq=dirpe0xb9q8qiLsFr0=vr0=vr0dc8meaabaqaciaacaGaaeqabaqabeGadaaakeaacuaH7oaBgaqcaaaa@2E70@ = 4.71*10^-22 ^and α^
 MathType@MTEF@5@5@+=feaafiart1ev1aaatCvAUfKttLearuWrP9MDH5MBPbIqV92AaeXatLxBI9gBaebbnrfifHhDYfgasaacH8akY=wiFfYdH8Gipec8Eeeu0xXdbba9frFj0=OqFfea0dXdd9vqai=hGuQ8kuc9pgc9s8qqaq=dirpe0xb9q8qiLsFr0=vr0=vr0dc8meaabaqaciaacaGaaeqabaqabeGadaaakeaacuaHXoqygaqcaaaa@2E5B@ = 2.78*10^-13 ^(see Figure 2b in Additional file 1). A total of 69 data points outside the dynamic range were excluded from further analyses (see Result 2.1 and Figure 2c in Additional file 1). To avoid over-fitting, only one of the three replicates, Replicate 1, was used for model development. Replicate 1 was chosen (907 data points) because it had the highest similarity to the other replicates (see Table 1 in Additional file 1); *i.e*., it was closest to the centroid of the replicate set.

**Figure 3:**
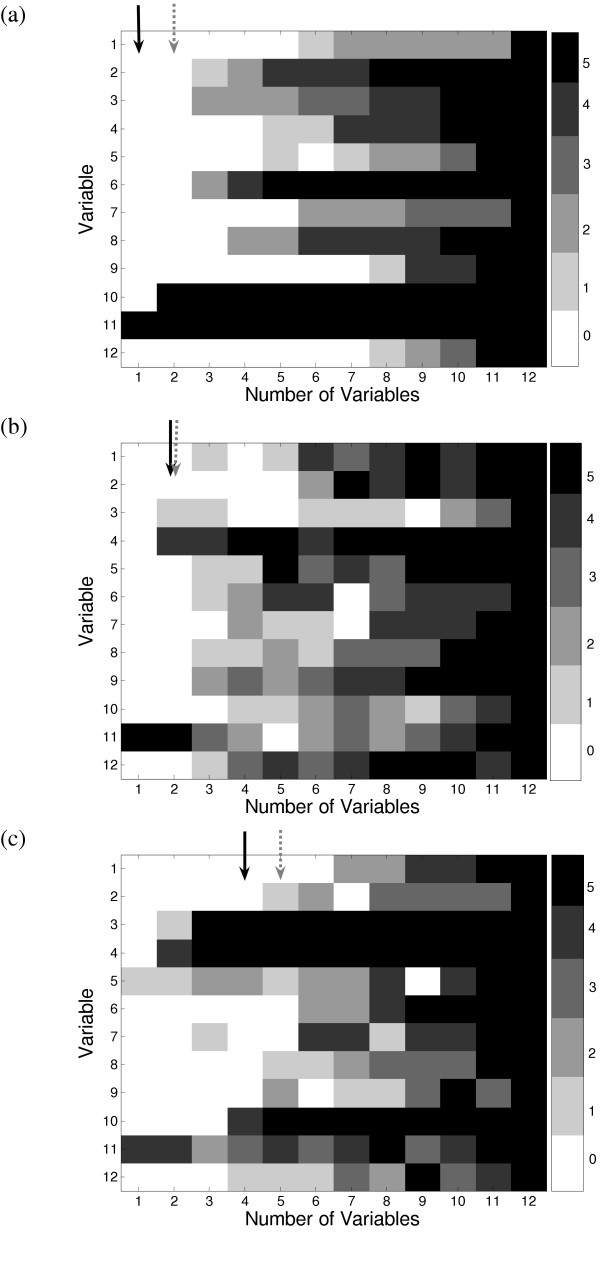
**Variables selected in five fold cross-validation (CV) for the models**. Variables (*X*_1 _to *X*_12_) are plotted versus model subset size (*p*). Counts of the selected variables in five-fold cross-validation for (a) multiple linear regressions (MLRs), (b) regression trees (RTs), and (c) artificial neural networks (ANNs) as subset size, *p*, increases from 1 to 12 along x-axis. The darker the color the more often a variable (y-axis) was selected for a model with a given number of independent variables (x-axis). Light-dotted and dark-solid arrows indicate the models with minimum errors and the most parsimonious models within one standard error of the minimum, respectively, as in Figure 2.

Twelve potential predictors were included in the model (see Methods, Table 1). The pairwise correlations between all pairs of variables and hybridization intensities (*X*_1 _to *X*_12 _and *TY*) were summarized in Result 2.2 in Additional file 1 (see Figure 3 in Additional file 1). As expected, some of the variables were correlated. The probe-target pairs with more most-contiguous-base-pairs (*X*_11_) and long overlaps (*X*_8_) often had higher intensities (*TY *> 6.5) than others (Figure 1).

### Multivariate models

Three multivariate methods, multiple linear regression (MLR), regression tree (RT) analysis, and feed-forward artificial neural network (ANN) analysis were performed to predict hybridization (for details see Methods). The results from these analyses were compared with that of the third-order polynomial regression, using percent identity (*X*_7_) as the sole predictor, as proposed by Xu and collaborators [11] [Equation (3) in Methods]. Five-fold cross-validation (CV) was performed to estimate the generalized errors [31] for all types of models so that the estimated errors were directly comparable. Models with all possible combinations of 12 potential predictors (4,095 combinations) were fitted and evaluated in each CV fold, and the model with the minimum sum of square errors was selected when *p *variables were included in the model (*p *= 1, 2, ..., 12). In the case of a closed-form solution for the model identification procedure (as in MLR), one-step CV was performed. Otherwise, two-step CV was performed: first-step CV to make decisions on the most appropriate internal model complexity and second-step CV to estimate the generalized errors of the final model (such as RTs and ANNs; for details see Methods).

#### Third-order polynomial regression (PR)

The third-order polynomial model using percent identity (*X*_*7*_) as the single predictor [11] was significant (*R*^2 ^= 0.31, *p *< 10^-4^). The polynomial terms were statistically significant, and the point estimates were β^0
 MathType@MTEF@5@5@+=feaafiart1ev1aaatCvAUfKttLearuWrP9MDH5MBPbIqV92AaeXatLxBI9gBaebbnrfifHhDYfgasaacH8akY=wiFfYdH8Gipec8Eeeu0xXdbba9frFj0=OqFfea0dXdd9vqai=hGuQ8kuc9pgc9s8qqaq=dirpe0xb9q8qiLsFr0=vr0=vr0dc8meaabaqaciaacaGaaeqabaqabeGadaaakeaacuaHYoGygaqcamaaBaaaleaacqaIWaamaeqaaaaa@2F77@ = -53.28, β^1
 MathType@MTEF@5@5@+=feaafiart1ev1aaatCvAUfKttLearuWrP9MDH5MBPbIqV92AaeXatLxBI9gBaebbnrfifHhDYfgasaacH8akY=wiFfYdH8Gipec8Eeeu0xXdbba9frFj0=OqFfea0dXdd9vqai=hGuQ8kuc9pgc9s8qqaq=dirpe0xb9q8qiLsFr0=vr0=vr0dc8meaabaqaciaacaGaaeqabaqabeGadaaakeaacuaHYoGygaqcamaaBaaaleaacqaIXaqmaeqaaaaa@2F79@ = 253.21, β^2
 MathType@MTEF@5@5@+=feaafiart1ev1aaatCvAUfKttLearuWrP9MDH5MBPbIqV92AaeXatLxBI9gBaebbnrfifHhDYfgasaacH8akY=wiFfYdH8Gipec8Eeeu0xXdbba9frFj0=OqFfea0dXdd9vqai=hGuQ8kuc9pgc9s8qqaq=dirpe0xb9q8qiLsFr0=vr0=vr0dc8meaabaqaciaacaGaaeqabaqabeGadaaakeaacuaHYoGygaqcamaaBaaaleaacqaIYaGmaeqaaaaa@2F7B@ = -365.11, β^4
 MathType@MTEF@5@5@+=feaafiart1ev1aaatCvAUfKttLearuWrP9MDH5MBPbIqV92AaeXatLxBI9gBaebbnrfifHhDYfgasaacH8akY=wiFfYdH8Gipec8Eeeu0xXdbba9frFj0=OqFfea0dXdd9vqai=hGuQ8kuc9pgc9s8qqaq=dirpe0xb9q8qiLsFr0=vr0=vr0dc8meaabaqaciaacaGaaeqabaqabeGadaaakeaacuaHYoGygaqcamaaBaaaleaacqaI0aanaeqaaaaa@2F7F@ = 173.35. The estimated CV error was 0.9981 (± 0.0889) [Equations (4) and (5) in Methods]. The residuals were examined with respect to the predictor, and no obvious pattern was detected to suggest any model violation.

#### Multiple linear regression (MLR)

**Figure 4:**
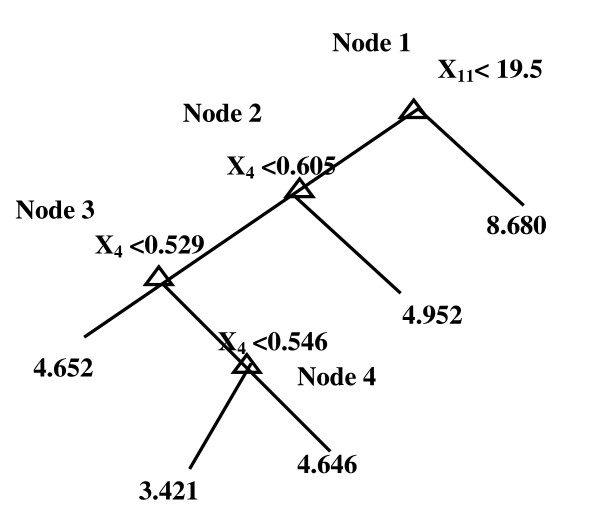
**Optimal regression tree**. Optimal regression tree with predictors [most contiguous base pair (X_11_) and target GC content (X_4_)] included in the model.

A total of 20,475 (= 4,095 × 5) multiple linear regression models [Equation (6) in Methods] were computed, and the model with the minimum sum of square errors at a given subset size *p *was selected (see Figure 4a in Additional file 1). The CV errors of all subset sizes were estimated (Figure 2a). The multiple linear regression with minimum CV errors (0.9123) contained two variables (Figure 2a). The most parsimonious model within one standard error of the minimum CV errors, the model with *p *= 1, was chosen [31]. Its only variable was the most contiguous base pairs (*X*_11_) (Figure 3a). The regression coefficients were estimated using the full dataset after the model subset size was decided. The regression model was significant (*R*^2 ^= 0.35, *p *< 10^-4^). The transformed hybridization increased 0.029 units as the most contiguous base pair increased by one unit. The residuals were examined, and no obvious pattern was detected to suggest model violation.

#### Regression tree (RT)

A total of 4,095 large trees was grown for each of the five CV training sets (for details see Methods and Methods 1.1 in Additional file 1). Each large tree was then pruned. The first-step CV was performed to compute the cost for each subtree. The smallest tree within one standard error of the minimum-cost subtree was selected [32]. The model with the minimum sum of square errors at a given subset size *p *was selected (see Figure 4b in Additional file 1). The generalized errors were estimated in the second-step CV (Figure 2b). The model with minimum CV errors was the model of subset size 2, and it was also the most parsimonious model within one standard error (Figures 2b). The models of subset size 2 were not all the same across the five CV training sets (Figure 3b), and the majority (four of the five) contained *X*_11 _(most contiguous base pairs) and *X*_4 _(target GC content). We therefore fitted the model using the entire dataset with *X*_11 _and *X*_4 _to derive the optimal regression tree. This subtree partitioned the feature space into five decision regions (Figure 4). Node 1 at the root is the most contiguous base pairs (*X*_11 _> 19.5), which can separate strong hybridizations from others. When there are more than 20 contiguous base pairs, the transformed hybridization intensities were stronger than 8.68 (Figure 4). The space became dichotomized three times (Nodes 2 to 4) after the first node, by the target GC content (*X*_4_). That is, target GC content influenced the hybridization levels in a nonlinear fashion. The residuals were examined, and no obvious pattern was detected.

#### Artificial Neural Network (ANN)

The first-step CV for early stopping was performed to select the appropriate number of hidden nodes to avoid overfitting for the 4,095 models in each training sets (see Figure 4c in Additional file 1). The model with the minimum sum of square errors at a given subset size *p *was selected (see Figure 4c in Additional file 1). The generalized errors were calculated in the second-step five-fold CV to decide the appropriate number of variables to retain in the models (Figure 2c). The model with minimum CV errors was of subset size 5 (CV error = 0.7487). The most parsimonious model within one standard error (0.067) was the model with four predictors (Figure 2c). The majority contained variables *X*_3 _(target length), *X*_4 _(target GC content), and *X*_10 _(target di-nucleotide distance), and *X*_11 _(most contiguous base pairs) (Figure 3c). Two exceptions were the models having *X*_11_replaced by *X*_5 _(Smith-Waterman score). This variable substitution is not surprising because *X*_5 _and *X*_11 _are linearly highly correlated (*r *= 0.98, *p *< 10^-165^). Interestingly, the rank correlation is much lower than the linear correlation (*r *= 0.14, *p *< 3.88*10^-5^). The target GC content and lengths influenced the hybridization intensities in a nonlinear fashion in addition to the effects of the most contiguous base pair. The residuals were examined with respect to the predictor, and no obvious pattern was detected.

### Model comparisons

**Figure 5:**
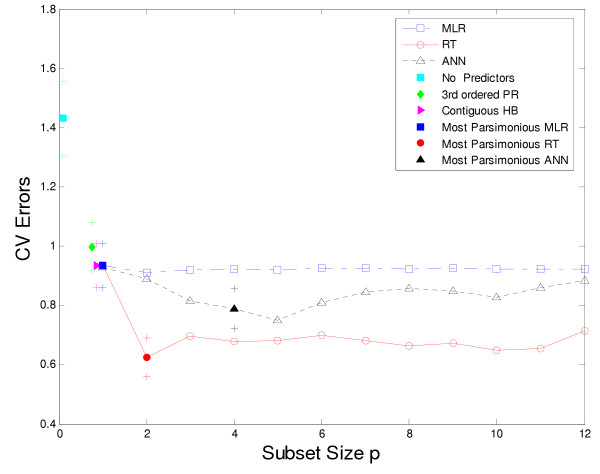
**Cross-validation errors of the multivariate models**. Cross-validation errors [Equation (4)] among the three multivariate models (MLR, RT, and ANN) and the third-order polynomial regression [11]. The chosen optimal model for each of the three multivariate methods is labeled with enlarged solid symbols with "+" indicating one standard error of the CV errors. The linear model using the most contiguous hydrogen bond (by treating GC as 3 hydrogen bonds and AT as 2 hydrogen bonds as used in Wren *et al*.; labeled as contiguous HB) had comparable performance as the linear model using most contiguous base pair as the sole predictor (MLR when *p *= 1). The cross-validated variance of *TY*, labeled as "no predictors", is 1.43 ± 0.13. Regression tree with two variables, *X*_11 _and *X*_4_, outperformed the other multivariate and univariate models.

Comparison of CV errors among models showed that the multivariate models were superior to the univariate third-order polynomial model proposed earlier [11], and indicated that more than one variable was important for hybridization prediction (Figure 5). Regression trees and artificial neural networks improved the prediction by including additional nonlinear effects (see Table 2 in Additional file 1). The CV correlation provides a summary measure of prediction quality [Equation (7) in Methods]. The selected regression tree using the most contiguous base pairs (*X*_11_) and target GC content (*X*_4_) outperformed all other chosen models (*R*_-*k*(*i*) _= 0.75, *p *< <10^-4^; see Table 2 in Additional file 1).

## Discussion

DNA microarrays are widely used for transcriptomic profiling, where the expression of thousands of genes is monitored simultaneously. The correct interpretation of all such microarray experiments depends on reliable and specific signal quantification.

We combined a systematic variable selection scheme with multiple competing multivariate models to improve current predictability of hybridization models for cDNA microarrays. Variable selection progression using five-fold cross-validation clearly showed that neither the sequence percent identity (*X*_7_), the variable identified in previous univariate studies [10-12], nor the E-value (*X*_6_), the variable heuristically used to measure hybridization potentials for arrays [26,33], was the most predictive independent variable. Instead, we found the most contiguous base pairs (*X*_11_) to be most predictive when only a single variable was selected (Figure 3). Prior to our final analysis using all 12 potential predictors, *X*_1 _to *X*_12_, we had performed a preliminary analysis using the first 10 potential variables, *X*_1 _to *X*_10_, for all three multivariate models with the same systematic search scheme. The results were fairly consistent with what we found using all 12 variables, with the noticeable exception that the most contiguous base pair, *X*_11_, was replaced by the Smith-Waterman alignment score, *X*_5_, for all three models, MLRs, RTs, and ANNs (see Figures 5 and 6 in Additional file 1). This variable substitution is to be expected because *X*_5 _and *X*_11 _are linearly highly correlated (*r *= 0.98, *p *< 10^-165^). The performance of the most parsimonious models for all methods of our final analyses, which included variable *X*_11_, was slightly improved over the preliminary analyses, which used variable *X*_5 _(see Tables 2 and 3 in Additional file 1). Although both ANNs and RTs do not have closed-form solutions, the consistent results yielded by the models using 10 or 12 variables showed the robustness of this method we used.

Our result showed that the most contiguous base pair (*X*_11_) and target GC content (*X*_4_) were the most predictive predictors in the selected regression tree (Figure 4), and it resonates with the finding by Wren *et al*. [13], but with significant improvements. Wren *et al*. only used one predictor, the most contiguous hydrogen bonds, in their model while we examined the relationships between all possible combinations of potential predictors and hybridization. They found that signals above background levels begin at ~45 hydrogen bonds (HBs) and become prominent after ~60 HBs [13]. As expected, the most contiguous hydrogen bond is highly correlated with the most contiguous base pair (*X*_11_) in our study (*r *= 0.9988, *p *≈ 0). The selected regression tree in our study showed that hybridizations were strong when more than 20 contiguous base pairs were found between probe and target pairs (Node 1 in Figure 4). Using the same hydrogen bond conversion (GC having 3 hydrogen bonds and AT having 2 hydrogen bonds), the hydrogen bond numbers for 20 base pairs segment are between 40 and 60. After separation at Node 1 (*X*_11_) in the regression tree, target GC content (*X*_4_) was found to influence hybridizations in a nonlinear fashion by further dichotomizing the decision space three times (Nodes 2 to 4 in Figure 4). Node 2 separated the second highest intensities with the remaining according to whether GC content exceeds 60%, supporting the intuition that targets having higher GC content have higher hybridization strength with probe sequences. The remaining two nodes divide the remaining space into three regions. The need of nonlinearities in hybridization model is not surprising because there is no straight forward prediction algorithm for prediction of secondary structure or folding energy for long sequences, such as the target sequences in our study. However, folding energy of sequences is generally correlated to GC content as illustrated by the high correlation found in probe GC content and estimated probe folding energy (see Figure 3 in Additional file 1). The nonlinear relationship between target GC content and hybridization may reflect the complex effects and interactions between secondary structure of target sequences and the hybridization between probe and target sequences for microarrays.

Predictability of the model could be improved in the future, for instance, by accounting for thermodynamic features, as it is sometimes done for oligonucleotide arrays [6-8]. Efforts are also under development to improve the computation speed for large dataset [34] and accommodate the constraints of unequal lengths between probe/target sequences and for long sequences in real world data [35].

Recently, after "jaw dropping" discordant results [36] among array platforms were reported [37], reproducibility across-platform has become a research topic of intense interest [38-42]. One of the contributing factors to the inconsistencies across platforms is thought to be due to the intrinsic differences of each array platforms [37]. The systematic multivariate approach proposed here can easily be applied to understand platform-specific hybridization processes, and this can potentially improve the comparability across platforms.

The major limitation of our model development is the use of a small dataset. At this point, the analysis of a relatively simple and small system seems to be the only way forward. Our proposed method should thus be seen as the first step toward understanding more fully the complexities surrounding cross-hybridization in other, larger systems. The hope behind our work is that scientists will begin to generate larger and more generalized datasets with hybridization between many targets and probes (see Figure 1a in Additional file 1), so that better and more widely applicable models may be developed in the near future.

## Conclusion

We proposed and validated a systematic strategy using multiple competing multivariate models to select critical sequence characteristics and quantify their relationship with hybridization on microarrays. The multivariate models outperformed the currently used univariate model in all cases. The most contiguous base pairs and the target GC content were found to be significant predictors of hybridization. Our systematic approach offers a quantitative method to correct for cross-hybridization signals on microarrays and shows the benefit of modeling nonlinear interdependencies between predictors and hybridization intensities.

## Methods

### Microarray data

A dataset of CYP450 PCR products spotted on microarrays [30] was used in this study. Thirty-one different DNAs from the CYP450 family (with lengths ranging from 500 to 1,200 bp) were hybridized individually with each of 31 arrays. Triplicates were generated, for a total of 93 arrays. Each array had 31 probes spotted at 1 μM. The probes were ~150 mer (ranging from 129–170 bp) PCR products, which corresponded to the 31 transcripts. The array manufacture details were described in [30]. Target/probe concentrations within a dynamic range were kept constant [30]. Other hybridization conditions (such as consistent buffer composition, salt concentration, 42°C in 50% forrnamide-based hybridization condition) in this study were consistent across experiments [30]. The hybridization intensities in our study can be viewed as the "conditional binding affinities" (*i.e*., binding affinities conditioned on a constant probe/target concentration, experimental temperature, etc.).

### Data transformation

Triplicate data were used to estimate the parameters *λ *and *α *in the generalized log transformation of the hybridization intensities [Equation (1)]. This transformation with slightly different parameterizations, was developed independently by two research groups [43,44]:

hλ(z)=ln(z+z2+λ),     (1)
 MathType@MTEF@5@5@+=feaafiart1ev1aaatCvAUfKttLearuWrP9MDH5MBPbIqV92AaeXatLxBI9gBaebbnrfifHhDYfgasaacH8akY=wiFfYdH8Gipec8Eeeu0xXdbba9frFj0=OqFfea0dXdd9vqai=hGuQ8kuc9pgc9s8qqaq=dirpe0xb9q8qiLsFr0=vr0=vr0dc8meaabaqaciaacaGaaeqabaqabeGadaaakeaacqWGObaAdaWgaaWcbaGaeq4UdWgabeaakiabcIcaOiabdQha6jabcMcaPiabg2da9iabbYgaSjabb6gaUjabcIcaOiabdQha6jabgUcaRmaakaaabaGaemOEaO3aaWbaaSqabeaacqaIYaGmaaGccqGHRaWkcqaH7oaBaSqabaGccqGGPaqkcqGGSaalcaWLjaGaaCzcamaabmaabaGaeGymaedacaGLOaGaayzkaaaaaa@44F5@

where z = y - α, and λ=σε2ση2
 MathType@MTEF@5@5@+=feaafiart1ev1aaatCvAUfKttLearuWrP9MDH5MBPbIqV92AaeXatLxBI9gBaebbnrfifHhDYfgasaacH8akY=wiFfYdH8Gipec8Eeeu0xXdbba9frFj0=OqFfea0dXdd9vqai=hGuQ8kuc9pgc9s8qqaq=dirpe0xb9q8qiLsFr0=vr0=vr0dc8meaabaqaciaacaGaaeqabaqabeGadaaakeaacqaH7oaBcqGH9aqpdaWcaaqaaiabeo8aZnaaDaaaleaacqaH1oqzaeaacqaIYaGmaaaakeaacqaHdpWCdaqhaaWcbaGaeq4TdGgabaGaeGOmaidaaaaaaaa@3897@.

This transformation is based on the expression model

yi=α+μ⋅eηi+εii=1,…,n     (2)
 MathType@MTEF@5@5@+=feaafiart1ev1aaatCvAUfKttLearuWrP9MDH5MBPbIqV92AaeXatLxBI9gBaebbnrfifHhDYfgasaacH8akY=wiFfYdH8Gipec8Eeeu0xXdbba9frFj0=OqFfea0dXdd9vqai=hGuQ8kuc9pgc9s8qqaq=dirpe0xb9q8qiLsFr0=vr0=vr0dc8meaabaqaciaacaGaaeqabaqabeGadaaakeaafaqabeqacaaabaGaemyEaK3aaSbaaSqaaiabdMgaPbqabaGccqGH9aqpcqaHXoqycqGHRaWkcqaH8oqBcqGHflY1cqWGLbqzdaahaaWcbeqaaiabeE7aOnaaBaaameaacqWGPbqAaeqaaaaakiabgUcaRiabew7aLnaaBaaaleaacqWGPbqAaeqaaaGcbaGaemyAaKMaeyypa0JaeGymaeJaeiilaWIaeSOjGSKaeiilaWIaemOBa4gaaiaaxMaacaWLjaWaaeWaaeaacqaIYaGmaiaawIcacaGLPaaaaaa@4B7B@

[45], where *y *represents the measured raw hybridization intensity, α is the background noise, μ is the true hybridization level, ε and η are normally distributed error terms with mean 0 and variances σε2
 MathType@MTEF@5@5@+=feaafiart1ev1aaatCvAUfKttLearuWrP9MDH5MBPbIqV92AaeXatLxBI9gBaebbnrfifHhDYfgasaacH8akY=wiFfYdH8Gipec8Eeeu0xXdbba9frFj0=OqFfea0dXdd9vqai=hGuQ8kuc9pgc9s8qqaq=dirpe0xb9q8qiLsFr0=vr0=vr0dc8meaabaqaciaacaGaaeqabaqabeGadaaakeaacqaHdpWCdaqhaaWcbaGaeqyTdugabaGaeGOmaidaaaaa@3135@ and ση2
 MathType@MTEF@5@5@+=feaafiart1ev1aaatCvAUfKttLearuWrP9MDH5MBPbIqV92AaeXatLxBI9gBaebbnrfifHhDYfgasaacH8akY=wiFfYdH8Gipec8Eeeu0xXdbba9frFj0=OqFfea0dXdd9vqai=hGuQ8kuc9pgc9s8qqaq=dirpe0xb9q8qiLsFr0=vr0=vr0dc8meaabaqaciaacaGaaeqabaqabeGadaaakeaacqaHdpWCdaqhaaWcbaGaeq4TdGgabaGaeGOmaidaaaaa@313A@, respectively, and *n *denotes sample size. The transformation not only agrees with the widely used log transformation [46], but also stabilizes the variance, satisfying the equal-variance assumption for linear models [47]. Maximum likelihood estimation implemented in the software package R [48] was used to estimate the parameters α and λ. The hybridization intensities used in the analyses were transformed according to the estimated form of Equation (1) and are denoted by *TY*. Even though triplicates were used for the estimation of α and λ, only one of the triplicates was used for model fitting and cross-validation so that estimates would not be overly optimistic.

### Potential predictors

Twelve potential predictors, reported to be important for predicting hybridization, were included in our study (Table 1). Probe/target sequence lengths and GC contents, variables *X*_1 _to *X*_4_, are important for hybridization [5,13,30]. Sequence alignment features are always thought to be important. For instance, sequence percent identity, *X*_7_, is considered the best predictor for cross-hybridization on cDNA microarrays, based on several univariate models [10-12]. Other alignment features were Smith-Waterman alignment score (*X*_5_), E-value (*X*_6_), and overlap length (*X*_8_). They were indicated as potential good predictors in univariate studies or used empirically for predicting hybridization [10,12,26,33]. The program *ssearch34*, [49,50], a rigorous and efficient implementation of the Smith-Waterman algorithm [51], was used to calculate these alignment features.

Secondary structures of sequences are important for hybridization interference, and the free energy for the 31 probe DNA sequences, *X*_9_, was estimated using *Mfold *[52]. The target sequences were long (many over 800 bp) so that the existing algorithm had no reasonable prediction performance for their folding energy or hybridization potential (cf. [52]). Thus, no prediction of the folding energy of the target transcripts was included in the model. One important feature to determine the hybridization potential between oligo sequences is the magnitude of pairwise base stacking of hybridization free energy by summing up all pairs of the free energy between neighboring two-base pairs, called the nearest-neighbor model [5,53,54]. There is no simple way to generalize this model for long and unequal-length sequences. Therefore, the standardized Euclidean distance between target-probe pairs, *X*_10_, using the alignment-free method with di-nucleotide word frequency [55] was used as a variable to mimic the empirical effect of nearest-neighborhood model for oligo sequences. Short segments of strong hybridization have been believed to be critical for predicting hybridization potentials [13,56]. As suggested by an anonymous reviewer, we included two more variables as potential predictors, *X*_11 _and *X*_12_, in our final analyses. The 'most contiguous base pairs between probe and target pairs' (or the length of identical substring) was included as variable *X*_11_. The most contiguous hydrogen bonds[13], considering GC having three hydrogen bonds and AT having two hydrogen bonds, would be an interesting variable to include. However, this variable is highly correlated to the most contiguous base pairs (r = 0.9988, p <10^-10^), and therefore, we included a more independent variable, the GC content of the most contiguous segment, as *X*_12_.

### Preliminary analysis

The pairwise linear and rank (Spearman) correlations between all 12 variables and the transformed hybridization intensities (*X*_1 _to *X*_12 _and *TY*) were examined. The correlations among triplicates were also examined to confirm the reproducibility and quality of the dataset.

### Multivariate models

Three multivariate methods, multiple linear regression (MLR), regression tree (RT) analysis, and feed-forward artificial neural network (ANN) analysis were performed to model hybridization. The comparative use of these three methods was to cover a range of possibilities that stretches from the computationally straightforward use and interpretation of multiple linear regression to computationally intensive and algorithmically intricate machine learning methods using artificial neural networks with early stopping and topology optimization [57]. Between these extremes, we also considered decision trees which iteratively dichotomize the complex domain based on different combinations of variables and correspondingly produce models that are easy to interpret [32]. The results from these analyses were compared with that of the third-order polynomial regression proposed by Xu and collaborators using percent identity as sole predictor [11] :

Y=β0+β1X7+β2X72+β3X73+ε.     (3)
 MathType@MTEF@5@5@+=feaafiart1ev1aaatCvAUfKttLearuWrP9MDH5MBPbIqV92AaeXatLxBI9gBaebbnrfifHhDYfgasaacH8akY=wiFfYdH8Gipec8Eeeu0xXdbba9frFj0=OqFfea0dXdd9vqai=hGuQ8kuc9pgc9s8qqaq=dirpe0xb9q8qiLsFr0=vr0=vr0dc8meaabaqaciaacaGaaeqabaqabeGadaaakeaacqWGzbqwcqGH9aqpcqaHYoGydaWgaaWcbaGaeGimaadabeaakiabgUcaRiabek7aInaaBaaaleaacqaIXaqmaeqaaOGaemiwaG1aaSbaaSqaaiabiEda3aqabaGccqGHRaWkcqaHYoGydaWgaaWcbaGaeGOmaidabeaakiabdIfaynaaDaaaleaacqaI3aWnaeaacqaIYaGmaaGccqGHRaWkcqaHYoGydaWgaaWcbaGaeG4mamdabeaakiabdIfaynaaDaaaleaacqaI3aWnaeaacqaIZaWmaaGccqGHRaWkcqaH1oqzcqGGUaGlcaWLjaGaaCzcamaabmaabaGaeG4mamdacaGLOaGaayzkaaaaaa@4D0A@

All multivariate model identification strategies proceeded as follows. Five-fold cross-validation (CV) was performed to estimate the generalized errors of the model [31]. The data were split into *k *(= 5) roughly equal-size parts. For the *k*^th ^part, the model was fitted to the other *k*-1 (= 4) parts of the data. Models with all possible combinations of variables (4,095 combinations) were fitted and evaluated, and the model with the minimum sum of square errors was selected when *p *variables were included in the model (*p *= 1, 2, ..., 12). The prediction error of the selected model was then calculated for the *k*^th ^part. A total of 20,475 models (5 × 4095) were trained over five folds. This approach may be viewed as five "CV training sets" and five "CV testing sets". The procedure was carried out for *k *= 1, 2,..., 5, and then the CV estimate of the prediction error (CV errors) was computed as

CV=1n∑i=1n(yi−y^i−k(i))2     (4)
 MathType@MTEF@5@5@+=feaafiart1ev1aaatCvAUfKttLearuWrP9MDH5MBPbIqV92AaeXatLxBI9gBaebbnrfifHhDYfgasaacH8akY=wiFfYdH8Gipec8Eeeu0xXdbba9frFj0=OqFfea0dXdd9vqai=hGuQ8kuc9pgc9s8qqaq=dirpe0xb9q8qiLsFr0=vr0=vr0dc8meaabaqaciaacaGaaeqabaqabeGadaaakeaacqWGdbWqcqWGwbGvcqGH9aqpdaWcaaqaaiabigdaXaqaaiabd6gaUbaadaaeWbqaaiabcIcaOiabdMha5naaBaaaleaacqWGPbqAaeqaaOGaeyOeI0IafmyEaKNbaKaadaqhaaWcbaGaemyAaKgabaGaeyOeI0Iaem4AaSMaeiikaGIaemyAaKMaeiykaKcaaOGaeiykaKYaaWbaaSqabeaacqaIYaGmaaaabaGaemyAaKMaeyypa0JaeGymaedabaGaemOBa4ganiabggHiLdGccaWLjaGaaCzcamaabmaabaGaeGinaqdacaGLOaGaayzkaaaaaa@4C55@

where *k*(*i*) is the part containing observation *i*, and y^i−k(i)
 MathType@MTEF@5@5@+=feaafiart1ev1aaatCvAUfKttLearuWrP9MDH5MBPbIqV92AaeXatLxBI9gBaebbnrfifHhDYfgasaacH8akY=wiFfYdH8Gipec8Eeeu0xXdbba9frFj0=OqFfea0dXdd9vqai=hGuQ8kuc9pgc9s8qqaq=dirpe0xb9q8qiLsFr0=vr0=vr0dc8meaabaqaciaacaGaaeqabaqabeGadaaakeaacuWG5bqEgaqcamaaDaaaleaacqWGPbqAaeaacqGHsislcqWGRbWAcqGGOaakcqWGPbqAcqGGPaqkaaaaaa@3518@ is the fitted value for observation *i*, computed with the *k*(*i*)^th ^part of the data removed. The estimate of the standard error of the CV error [58] is

SE(CV)=s2n=1n=1∑i=1n[(yi−y^i−k(i))2−CV]2n.     (5)
 MathType@MTEF@5@5@+=feaafiart1ev1aaatCvAUfKttLearuWrP9MDH5MBPbIqV92AaeXatLxBI9gBaebbnrfifHhDYfgasaacH8akY=wiFfYdH8Gipec8Eeeu0xXdbba9frFj0=OqFfea0dXdd9vqai=hGuQ8kuc9pgc9s8qqaq=dirpe0xb9q8qiLsFr0=vr0=vr0dc8meaabaqaciaacaGaaeqabaqabeGadaaakeaacqWGtbWucqWGfbqrcqGGOaakcqWGdbWqcqWGwbGvcqGGPaqkcqGH9aqpdaGcaaqaamaalaaabaGaem4Cam3aaWbaaSqabeaacqaIYaGmaaaakeaacqWGUbGBaaaaleqaaOGaeyypa0ZaaOaaaeaadaWcaaqaamaalaaabaGaeGymaedabaGaemOBa4Maeyypa0JaeGymaedaamaaqahabaGaei4waSLaeiikaGIaemyEaK3aaSbaaSqaaiabdMgaPbqabaGccqGHsislcuWG5bqEgaqcamaaDaaaleaacqWGPbqAaeaacqGHsislcqWGRbWAcqGGOaakcqWGPbqAcqGGPaqkaaGccqGGPaqkdaahaaWcbeqaaiabikdaYaaakiabgkHiTiabdoeadjabdAfawjabc2faDnaaCaaaleqabaGaeGOmaidaaaqaaiabdMgaPjabg2da9iabigdaXaqaaiabd6gaUbqdcqGHris5aaGcbaGaemOBa4gaaaWcbeaakiabc6caUiaaxMaacaWLjaWaaeWaaeaacqaI1aqnaiaawIcacaGLPaaaaaa@60D1@

In the case of a closed-form solution (*i.e*., MLR), one-step CV was performed to estimate the generalized errors. For models that use CV to make decisions on the most appropriate internal model complexity in the first step, a second-step CV was used to estimate generalized errors. This step for regression trees and ANNs ensured that the generalized errors were estimated from data outside those training data that were used to fit (train/validate) the model in the first-step CV. The resulting CV errors were compared with those estimated by one step CV errors of MLR. The most parsimonious model within one standard error of the model with the minimum CV error was chosen [31] for each of the three multivariate methods. CV residuals of the selected models were examined with respect to the predictors to assess model assumptions.

#### Multiple linear regression (MLR)

The simplest relationship between the predictors and the hybridization intensities is linear:

*Y *= β_0 _+ β_1_*X*_1 _+ β_2_*X*_2 _+ ... + β_*p*_*X*_*p *_+ ε.     (6)

The appropriate number of variables, *p*, was determined using CV errors [Equation (4)] and its estimated standard errors [Equation (5)].

#### Regression tree (RT)

A regression tree, also known as CART (classification and regression tree), represents a multistage decision process, where a binary decision is made at each stage [32] to partition the *d*-dimensional space into smaller and smaller regions [58]. Three standard steps for regression tree modeling are growing a large tree, pruning, and finally selection of a subtree tree based on CV [32,58]. More details are summarized in Method 1.1 in Additional file 1. We performed a fourth step, namely second-step CV, to estimate generalized errors, using the data external to the training-testing data set used for the first-step CV in earlier steps. This second-step CV yielded an estimate of generalized errors, and determined the appropriate number of variables to retain in the models.

#### Artificial Neural Network (ANN)

An ANN is a two-stage nonlinear regression or classification method [31]. It identifies arbitrary multiparametric functions directly from experimental data as universal approximators [59]. The first-stage nonlinear regression is between the input predictors and the hidden layer, and the second-stage regression is between the hidden layer and the response variable.

We applied a series of measures to avoid potential pitfalls associated with ANN, such as model overfitting, input scaling problems, arbitrary numbers of hidden nodes, and multiple minima [31]. In our study, one hidden layer was used because it has been shown to be sufficient for approximating all functional forms [59]. All predictors were scaled between 0 and 1 in the feed-forward ANN to eliminate scaling effects. First-step CV for early stopping was performed to select an appropriate number of hidden nodes to avoid model overfitting [57]. In a similar manner to our treatment of regression trees, a second-step CV was performed with the ANN to decide the appropriate number of variables to retain. More details on ANN model fitting and topology optimization can be found in a comprehensive review [57].

#### Model performance

The estimates of CV errors [Equation (4)] were compared among three multivariate models and the third-order polynomial model. Furthermore, the CV correlation, calculated as

R−k(i)2=r2(yi,y^i−k(i)),     (7)
 MathType@MTEF@5@5@+=feaafiart1ev1aaatCvAUfKttLearuWrP9MDH5MBPbIqV92AaeXatLxBI9gBaebbnrfifHhDYfgasaacH8akY=wiFfYdH8Gipec8Eeeu0xXdbba9frFj0=OqFfea0dXdd9vqai=hGuQ8kuc9pgc9s8qqaq=dirpe0xb9q8qiLsFr0=vr0=vr0dc8meaabaqaciaacaGaaeqabaqabeGadaaakeaacqWGsbGudaqhaaWcbaGaeyOeI0Iaem4AaSMaeiikaGIaemyAaKMaeiykaKcabaGaeGOmaidaaOGaeyypa0JaemOCai3aaWbaaSqabeaacqaIYaGmaaGccqGGOaakcqWG5bqEdaWgaaWcbaGaemyAaKgabeaakiabcYcaSiqbdMha5zaajaWaa0baaSqaaiabdMgaPbqaaiabgkHiTiabdUgaRjabcIcaOiabdMgaPjabcMcaPaaakiabcMcaPiabcYcaSiaaxMaacaWLjaWaaeWaaeaacqaI3aWnaiaawIcacaGLPaaaaaa@4AB4@

provides a summary measure of prediction quality.

## Authors' contributions

JSA supervised the entire project and implemented ANNs. YAC performed the computational model development and implementation under the supervision of XL, EHS, EOV and JSA. YAC drafted the manuscript. CCC and KP provided the data and assistance with biological interpretations of the parametric sensitivities observed. WX proposed the third-order polynomial regression and provided biological insights. All authors contributed to the writing and revision of the manuscript and approved the final manuscript.

## Supplementary Material

Additional File 1 Supplements include additional methods, results, tables, and figures. Click here for file

## Figures and Tables

**Table 1:** List of covariates included in the model and method/algorithm of calculation.

Covariate	Description	Method
X_1_	Probe sequence length	count
X_2_	Probe GC content (%)	count
X_3_	Target length	count
X_4_	Target GC content (%)	count
X_5_	Smith-Waterman score	alignment (SW*)
X_6_	E-value	alignment (SW*)
X_7_	Percent identity	alignment (SW*)
X_8_	Overlap length (base pair)	alignment (SW*)
X_9_	Free energy for probe DNA folding**	Mfold [52]
X_10_	Standardized Euclidian distance	[55]
X_11_	Most contiguous base pairs	customized method
X_12_	GC content of the most contiguous segment	count

*SW is the abbreviation for the Smith-Waterman alignment algorithm.

**For ease of computation and interpretation, all covariates are shown as positive. *X*_9 _is minus ΔG, so its values are positive.
