# Supplementary material for: A multivariate prediction model for microarray cross-hybridization
Source: BMC Bioinformatics. 2006 Mar 1;7:101. doi: 10.1186/1471-2105-7-101 (PMC1409802; doi:10.1186/1471-2105-7-101)
Supplement: Additional File 1 — Supplements include additional methods, results, tables, and figures. [file 1471-2105-7-101-S1.DOC]

# Additional File 1 - Supplements

# 1 Supplementary Methods

## 1.1 Three standard steps for regression tree modeling

First, a large tree, *T_max_*, was grown. The total within-node sum-of-squares is given by

where *T* denotes the regression tree, *t* denotes a node in the tree, is the set of terminal nodes in the tree, (*x_i_*, *y_i_*) is one observation (case), and *n*(*t*) represents the total number of observations in that node. At each stage, the splitting variables and splitting points were decided automatically based on maximizing the change in the within-node sum-of-squares at node *t* using split *s*, given by

where *t_L_* and *t_R_* are the left and right child of the node.

Ideally, we could grow the tree until there is only one observation in each node at the end, and the error term would be zero for the whole tree (*R* (*T*) = 0). However, this tree would not necessarily fit new data well because there is a trade-off between complexity and generalization of the model. Therefore, the second stage was tree pruning based on the cost-complexity criterion

(s1)

where $\alpha$, the tuning (or complexity) parameter, is positive [1]. The cost-complexity measure, $C_{\alpha}(T)$, is a linear combination of the cost of the tree and its complexity. The cost of the regression tree, *R* (*T*), is the same as the squared error terms in other types of models (*e.g.*, linear regression or neural networks). For each $\alpha$; the idea is to find the subtree *T*$\alpha$ *T_max_* that minimizes the criterion in Equation (s1). The first-step CV was performed to compute the costs for all subtrees, and standard errors were estimated. The first-step CV errors were used to select the subtree with appropriate size. The third step was to select the best-pruned subtree such that the complexity of the tree and the error, *R* (*T*), were both minimized. The smallest tree within one standard error of the minimum-cost subtree was selected as the best tree [1].

# 2 Supplementary Results

## 2.1 Data points outside dynamic range were excluded for model development

Two clearly separated distributions of the hybridization intensities after transformation were observed (Supplementary Figure 2c). The values in the negative domain, with *TY* between –24 and –25, contained only 69 data points. The values in the positive domain, ranging between 0 and 11, contained the vast majority of the data (2,721 data points). It was apparent that no hybridization occurred in the population of the negative domain. Therefore, these 69 data points were excluded from further analyses.

## 2.2 Pairwise relationships between all 12 predictors and the transformed hybridization intensities (X_1_ to X_12_ and TY).

Some variables were correlated as expected (Figure 3a in the Supplements). The Smith-Waterman score (*X*_5_) indicated significant linear correlation with the hybridization levels (*R* = 0.58, *p* = 10^-83^) (Figures 2b-c in the Supplements) and *X*_11_ (*r* = 0.98, *p* < 10^-165^). Positive correlation between percent identity (*X*_7_) and *TY* was also observed. The results of the nonparametric Spearman correlation coefficient showed that four alignment features (*X*_5_ - *X*_8_) were correlated as expected, and that probe GC content (*X*_2_) and probe folding free energy (*X*_9_) were positively correlated (Figures 3b-e in the Supplements).

# 3 Supplementary Figures

Suppl. Figure 1. Graphic illustration of hybridization process on expression arrays.

(a)  Hybridization potentially occurs between multiple targets and multiple probes. *T_i_* denotes target *i*, where *i* = 1,2,…,*M*. *P_j_* denotes probe *j*, where *j* = 1, 2, ..., *N*. We are interested in predicting the hybridization *y_ij_* between *T_i_* and *P_j_*. (b) To study this complex phenomenon, we first study a simplified system with hybridization occurs between one target and multiple probe sequences spotted on the arrays.

| a. | b. |
| --- | --- |
|  |  |

**Suppl. Figure 2a**. Generalized log transformed (glog) data based on the triplicates of 31 target sequences (on 93 slides). $TY_{i}$=$\ln((Y_{i}-\alpha)+\sqrt{(Y_{i}-\alpha)^{2}+\lambda})$. The estimated parameters are $\hat{\lambda}=$ 1.39*10^-20^ and $\hat{\alpha}=$1.79*10^-12^. Hybridization intensities of target 17 in all three replicates were consistently lower than others, which suggested potential systematic errors, such as low target concentration in the sample.

|  |
| --- |

**Suppl. Figure 2b**. Generalized log transformed (glog) data based on the triplicates of 30 target sequences (on 90 slides) after excluding the potentially problematic data from target 17 (on slides 49-51). $TY_{i}$=$\ln((Y_{i}-\alpha)+\sqrt{(Y_{i}-\alpha)^{2}+\lambda})$. The estimated parameters are$\hat{\lambda}=$ 4.71*10^-22^ and $\hat{\alpha}=$2.78*10^-13^.

|  |
| --- |

**Suppl. Figure 2c**. Two separate distributions of expression data were observed after transformation. One of these, on the far left with 69 data points, was apparently far below the dynamic range. That is, no hybridization had occurred. The population on the right contained the majority of the data and offered data spanning a range from non-hybridization to cross-hybridization and specific-hybridization.

|  |
| --- |

**Suppl. Figure 3a.** Pairwise relationships between the predictors (*X*_1_-*X*_12_; columns and rows 1 to 12) and the transformed expression intensities (*TY*; column and row 13). The diagonal shows the distribution of each variable.

|  |
| --- |

**Suppl. Figures 3b & c.** Pair-wise linear correlation coefficients and their associated p-value between 13 variables [(*X*_1_-*X*_12_; columns and rows 1 to 12) and the transformed expression intensities (*TY*; column and row 13)]. The most contiguous base pairs showed significant correlation with the expression level (*R* = 0.59, *p* = 2.9 *10^-86^). Similar observations were made for the Smith-Waterman score (*X*_5_), percent identity (*X*_7_) and overlap length (*X*_8_) with lower correlation coefficients. Linear correlation analyses showed correlation pattern between 5 alignment variables (*X*_5_ –*X*_8_, and *X*_11_), and the correlation between probe GC content (*X*_2_) and the predicted folding energy (*X*_9_).

| Pair-wise linear correlation coefficients | Their associated p-values |
| --- | --- |

**Suppl. Figures 3d & e**. Pair-wise Spearman correlation coefficients and their associated p-values between 13 variables [(*X*_1_-*X*_12_; columns and rows 1 to 12) and the transformed expression intensities (*TY*; column and row 13)]. Nonparametric correlation analyses showed a similar but clearer correlation pattern between 5 alignment variables (*X*_5_ –*X*_8_ , and *X*_11_), and between probe GC content (*X*_2_) and the predicted folding energy (*X*_9_).

| Pair-wise Spearman correlation coefficients | Their associated p-values |
| --- | --- |

**Suppl. Figure 4a.** Training errors (SSE/sample size of the first CV training set) for the 4,095 multiple linear regression (MLR) models (with all possible combinations of variables) in the first “CV training set”. The model with the minimum error at a given model subset size *p* was chosen (referred to as the minimum training errors in Figure 2).

|  |
| --- |

**Suppl. Figure 4b.** After the first-step cross-validation, the errors (SSE/sample size of first CV training set) were estimated for the 4,095 regression tree (RT) models (with all possible combinations of variables) in the first CV training set. These errors were comparable to the training errors for MLR. The model with the minimum error at a given model subset size *p* was chosen (referred to as the minimum training errors in Figure 2).

|  |
| --- |

**Suppl. Figure 4c.** After the first-step cross-validation, the errors (SSE/sample size of first CV training set) were estimated for the 4,095 models of all possible combinations of variables in the first CV training set for the feed-forward artificial neural network (ANN). These errors were comparable to the training errors for MLR. The model with the minimum error at a given model subset size *p* was chosen (referred to as the minimum training errors in Figure 2).

|  |
| --- |

Suppl. Figure 5.  Training and cross-validation (CV) errors of the multivariate models when only the first 10 potential predictors, *X*_1_ to *X*_10_, were included for preliminary model development.

Minimum training errors (solid circles) of (a) multiple linear regressions (MLRs), (b) regression trees (RTs), and (c) artificial neural networks (ANNs) in the first CV training set decreased, while the CV errors [open squares; Equation (4)] reached the minimum (light-dotted arrows) at the subset size of 3 in (a), 2 in (b), and 4 in (c). The most parsimonious model (dark-solid arrows) within one standard error of the model with the minimum error was the model with 1 predictor for (a), 2 predictors for (b) and 3 predictors for (c). (The cross-validated variance of TY, for reference, is 1.43 ± 0.13).

| (a) |  |
| --- | --- |
| (b) |  |
| (c) |  |

Suppl. Figure 6.  Variables selected from *X*_1_ to *X*_10_ in five fold cross-validation (CV) for preliminary model developments.

Ten preliminary variables (*X*_1_ to *X*_10_) are plotted versus model subset size (*p*). Counts of the selected variables in five-fold cross-validation for (a) multiple linear regressions (MLRs), (b) regression trees (RTs), and (c) artificial neural networks (ANNs) as subset size, *p*, increases from 1 to 10 along x-axis. The darker the color the more often a variable (y-axis) was selected for a model with a given number of independent variables (x-axis). Light-dotted and dark-solid arrows indicate the models with minimum errors and the most parsimonious models within one standard error of the minimum, respectively, as in Supplementary Figure 5.

| (a) |  |
| --- | --- |
| (b) |  |
| (c) |  |

# 4 Supplementary Tables

**Suppl. Table 1.** Spearman correlations among the three replicates of hybridization data were high (the linear correlation coefficients ± 95% confidence intervals are listed in parenthesis).

| Replicates | 1 | 2 | 3 |
| --- | --- | --- | --- |
| 1 | 1 | 0.97 (0.99 ± 0.0012) | 0.97 (0.98 ± 0.0023) |
| 2 |  | 1 | 0.94 (0.98 ± 0.0022) |
| 3 |  |  | 1 |

**Suppl. Table 2.**Performance comparison among the four “most parsimonious” models selected when all 12 potential predictors were used for model development (described in Results). Cross-validation correlation coefficient was defined in Equation (7) of Methods.

| Models | CV errors (± se CV) | Variables | Cross-validation R ($R_{-k(i)}$) | P-value |
| --- | --- | --- | --- | --- |
| PR | 1.00 (± 0.09) | *X*_7_, *X*_7_^2^, *X*_7_^3^ | 0.55 | 5.17*10^-73^ |
| MLR | 0.94 (± 0.07) | *X*_11_ | 0.59 | 8.24*10^-86^ |
| RT | 0.62 (± 0.06) | *X*_4_, *X*_11_ | 0.75 | 1.84*10^-165^ |
| ANN | 0.79 (± 0.07) | *X*_3_, *X*_4_, *X*_10_, *X*_11_ | 0.67 | 2.55*10^-120^ |

**Suppl. Table 3.**Performance comparison among the four “most parsimonious” models selected when 10 potential predictors, *X*_1_ to *X*_10_, were used for preliminary model development (described in Discussion). Cross-validation correlation coefficient was defined in Equation (7) of Methods.

| Models | CV errors (± se CV) | Variables | Cross-validation R ($R_{-k(i)}$) | P-value |
| --- | --- | --- | --- | --- |
| PR | 1.00 (± 0.09) | *X*_7_, *X*_7_^2^, *X*_7_^3^ | 0.55 | 5.17*10^-73^ |
| MLR | 0.95 (± 0.07) | *X*_5_ | 0.58 | 4.41*10^-83^ |
| RT | 0.70 (± 0.07) | *X*_4_, *X*_5_ | 0.72 | 1.62*10^-145^ |
| ANN | 0.86 (± 0.08) | *X*_3_, *X*_4_, *X*_5_ | 0.64 | 1.35*10^-103^ |

# 5 Supplementary References

1. Breiman L, Friedman JH, Olshen R, Stone C**: Classification and regression tr**ee. New York: Wadsworth Inc; 1984.
